# A three-factor plasma diagnostic model of human T -cell leukemia virus-1 associated myelopathy

**DOI:** 10.1186/1742-4690-12-S1-O18

**Published:** 2015-08-28

**Authors:** Makoto Ishihara, Natsumi Araya, Tomoo Sato, Naomi Saichi, Risa Fujii, Yoshihisa Yamano, Koji Ueda

**Affiliations:** 1 Division of Biosciences, Functional Proteomics Center, Graduate School of Frontier Sciences, The University of Tokyo, Tokyo, Japan; 2 Department of Rare Diseases Research, Institute of Medical Science, St. Marianna University School of Medicine, Kawasaki, Japan

## 

HTLV-1 associated myelopathy/tropic spastic paraparesis (HAM/TSP) is induced by chronic inflammation in spinal cord due to HTLV-1 infection. Cerebrospinal fluid (CSF) neopterin or proviral load are clinically measured as disease grading biomarkers, however, they are not exactly specific to HAM/TSP. Therefore, we aimed to identify HAM/TSP-specific biomarker molecules and establish a novel less-invasive plasma diagnostic model for HAM/TSP. Proteome-wide quantitative profiling of CSFs from 6 asymptomatic HTLV-1 carriers (AC) and 51 HAM/TSP patients was performed. Quantitative analysis for 1,871 nonredundant CSF proteins identified from 57 individuals defined 14 CSF proteins showing significant correlation with Osame's motor disability score (OMDS). The 14 severity grade biomarker proteins were further examined plasma ELISA assays (n = 71). This results confirmed secreted protein acidic and rich in cysteine (SPARC) and vascular cell adhesion molecule-1 (VCAM-1) demonstrated the same correlations in plasma (R = −0.373 and R = 0.431, respectively). Finally, we constructed three-factor logistic regression model and evaluated the diagnostic power using 105 plasma samples. In this training set, we constructed a HAM/TSP diagnostic model using SPARC, VCAM1, and viral load. Sensitivity and specificity to diagnose HAM/TSP patients from AC (AC vs. OMDS 1–11) were 85.3% and 81.1%, respectively. Importantly, this model could be also useful for determination of therapeutic intervention point (OMDS 1–3 + AC vs. OMDS 4–11), exhibiting 80.0% sensitivity and 82.9% specificity. We propose a novel less-invasive diagnostic model for early detection and clinical stratification of HAM/TSP.

